# Advanced Modular Honeycombs with Biomimetic Density Gradients for Superior Energy Dissipation

**DOI:** 10.3390/biomimetics10040221

**Published:** 2025-04-03

**Authors:** Yong Dong, Jie He, Dongtao Wang, Dazhi Luo, Yanghui Zeng, Haixia Feng, Xizhen You, Lumin Shen

**Affiliations:** 1 College of Intelligent Manufacturing and Mechanical Engineering, Hunan Institute of Technology, Hengyang 421002, China; 1997000315@hnit.edu.cn (Y.D.); 2016002080@hnit.edu.cn (J.H.); 2015001995@hnit.edu.cn (L.S.); 2 School of Vehicle Engineering, Hunan Automotive Engineering Vocational University, Zhuzhou 412009, China; 3 Zhuzhou Elite ElectroMechanical Co., Ltd., Zhuzhou 410004, China; luodazhi@nfelite.com (D.L.); zengyanghui@nfelite.com (Y.Z.); 4 Business School, Shanghai Sanda University, Shanghai 201209, China; wutong@sandau.edu.cn; 5 Beijing New Energy Vehicle Co., Ltd., Beijing 102606, China; youxizhen@baicgroup.com.cn

**Keywords:** modular honeycomb, enhanced mechanical property, out-plane, energy absorption

## Abstract

The honeycomb configuration has been widely adopted in numerous sectors owing to its superior strength-to-weight ratio, rigidity, and outstanding energy absorption properties, attracting substantial academic attention and research interest. This study introduces a biomimetic modular honeycomb configuration inspired by the variable-density biological enhancement characteristics of tree stem tissues. This study examined the out-of-plane compressive behavior and mechanical characteristics of modular honeycomb structures. A numerical model of the modular honeycomb was constructed utilizing finite element technology, enabling simulation studies at varying impact velocities. The improved weight-bearing and impact-absorbing properties of modular honeycomb structures are investigated using theoretical analysis and computer simulations. It also scrutinizes the effects of boundary and matching conditions on the honeycomb’s performance. The results indicate that adjusting the thickness of the walls in both the matrix honeycomb and sub-honeycomb structures can substantially improve their resistance to low-velocity out-of-plane compression impacts. Furthermore, the energy absorption capacity of modular honeycombs during high-velocity impacts is significantly influenced by multiple factors: the impact velocity, the density of the honeycomb structure, and the distribution of wall thickness within the sub-honeycomb and the primary honeycomb matrix. Notably, the modular honeycomb with an optimally designed structure demonstrates superior high-speed impact resistance compared to conventional honeycombs of equivalent density. These insights underscore the potential for advanced honeycomb designs to further advance material performance in structural applications.

## 1. Introduction

The demand for high-performance energy-absorbing materials in the engineering field is growing at an unprecedented rate [1,2,3]. These materials must meet increasingly stringent engineering requirements and achieve lightweight standards while ensuring structural safety. Among the many novel materials, periodic multi-cellular structures have gradually become the focus of research and application due to their unique topological structures and exceptional energy absorption capabilities [3,4,5,6]. These structures are three-dimensional materials composed of multiple repeating units, typically arranged in a regular pattern, forming a highly ordered engineering material. A typical example of a multi-cellular structure is the honeycomb structure, which consists of hexagonal cells arranged in a two-dimensional plane and extended perpendicular to that plane. These interconnected cells confer the characteristic high porosity and low density to honeycombs. Furthermore, this structure imparts exceptional specific stiffness and strength and superior energy absorption capabilities [7,8].

Honeycomb structures exhibit sustained plateau stress when subjected to impact, which facilitates the conversion of kinetic energy into plastic deformation energy, effectively absorbing and dissipating the impact force and preserving the structural integrity. Honeycomb structures have garnered substantial attention across numerous engineering disciplines, owing to their minimal weight and exceptional capacity for energy absorption, such as transportation [9], aerospace [10,11], packaging protection [12,13], and military defense [14]. For instance, in the automotive industry, honeycomb structures are used to manufacture energy-absorbing crumple zones to enhance vehicle safety during collisions [15]; in aerospace, they are used to create lightweight yet strong wing and satellite components to reduce weight and improve fuel efficiency [16]; and in packaging, honeycomb structures serve as cushioning materials to protect fragile items from damage during transportation [17]. Additionally, honeycomb structures show broad application potential in construction [18], sports equipment [19,20,21], and medical devices [22,23,24].

However, despite the advantageous lightweight and energy-absorbing characteristics of honeycomb structures, the enhancement of their specific energy absorption capacity remains a persistent objective in the fields of science and engineering, as it is essential to meet the escalating demands of engineering applications. Therefore, further research on the energy-absorbing characteristics of honeycomb structures is particularly urgent [25,26,27]. Recent comprehensive studies [28,29,30,31,32,33] have systematically reviewed the development of innovative configurations, including filled-type, embedded, tandem, hierarchical, and auxetic honeycombs, providing critical insights into their geometric optimization, mechanical enhancement, and dynamic energy absorption mechanisms. The design and optimization of honeycomb structures involve multiple disciplines, such as materials science [34], mechanics, and manufacturing processes [35]. Researchers are continuously exploring new design concepts and manufacturing technologies to enhance honeycomb structures’ performance further. For example, adjusting the geometric parameters of the honeycomb, such as wall thickness, cell size, and unit shape, can optimize its energy-absorbing characteristics [36,37]. Moreover, material selection also significantly affects the performance of honeycomb structures, as different materials provide varying degrees of strength and toughness, influencing their energy absorption effects [38,39,40,41].

The integration of bio-inspired principles derived from natural systems into the performance-enhanced design of honeycomb structures constitutes a significant research frontier [42]. In natural trees, the density across their cross-sections exhibits complex variation patterns [43]. From the perspective of annual ring formation, earlywood has lower density due to larger vessel lumens and thinner fiber cell walls, whereas latewood shows significantly higher density due to narrower vessel lumens and thickened fiber cell walls. Consequently, tree density exhibits periodic fluctuations across annual rings, with latewood density within a single ring being higher than that of earlywood [44]. From the viewpoint of species-specific anatomical characteristics, certain conifers display higher density in the heartwood than in the sapwood. In contrast, tropical hardwoods exhibit higher density near the bark due to continuous thickening of fiber cell walls in mature outer growth rings [45]. This study integrates both the radial density gradient patterns (from bark to pith) and the topological configuration of honeycomb structures to design a honeycomb block with locally varying wall thicknesses. These blocks simulate radial density gradients through modulated wall thicknesses. However, constrained by the porous nature of honeycomb structures and manufacturing feasibility, the stepwise thickening of cell walls and the stepwise density variations caused by annual ring transitions have not been replicated in the design. By assembling these radially thickness-modulated honeycomb blocks, a novel biomimetic modular honeycomb structure is proposed. The central idea is to form a variable-density structure that can optimize energy absorption and load-bearing capabilities. This biomimetic approach not only leverages the inherent advantages of honeycomb structures but also introduces new design parameters that can be tailored for specific applications.

This investigation integrates theoretical methodologies and finite element analysis to examine modular honeycombs’ out-of-plane load-bearing capacity and energy absorption enhancement mechanisms. It also evaluates the influence of boundary conditions and structural compatibility on their performance. These studies lay the foundation for applying modular honeycombs in energy absorption. The research presented herein provides novel insights and methodologies for enhancing the energy absorption efficiency of honeycomb structures and developing new honeycomb materials with superior mechanical properties, thereby advancing the field of high-performance mechanical materials.

## 2. Design and Method

### 2.1. Modular Honeycomb Design

**Figure 1:**
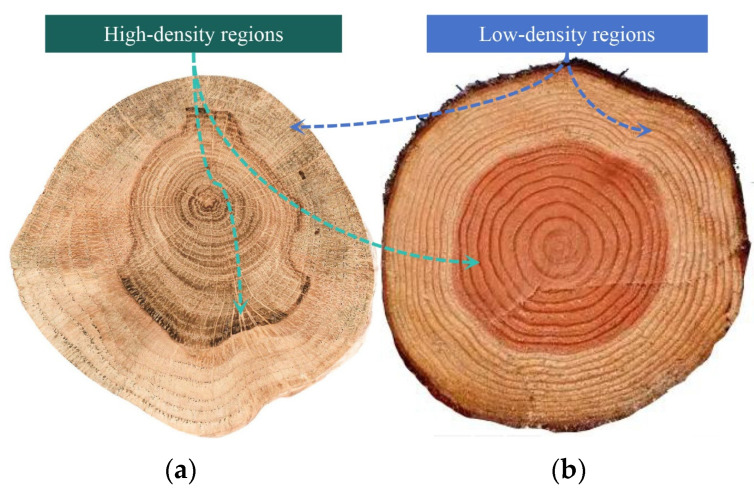
The phenomenon of variable density in tree stems: (**a**) oak and (**b**) pine.

The density across tree cross-sections typically exhibits non-uniform distribution. For instance, the wood density of Juglans nigra [46] and Zhongshanshan 118 [47] initially increases slightly from the pith outward and then gradually declines, resulting in higher density in heartwood compared to sapwood. Similarly, as shown in Figure 1, oak (Figure 1a) and pine (Figure 1b) also demonstrate that the central xylem regions exhibit higher density than peripheral zones. Building upon this natural phenomenon of radial gradient density distribution in trees rather than uniform patterns, a variable-density reinforcement strategy for cellular porous materials is developed, aiming to enhance the mechanical strength of honeycomb structures.

**Figure 2:**
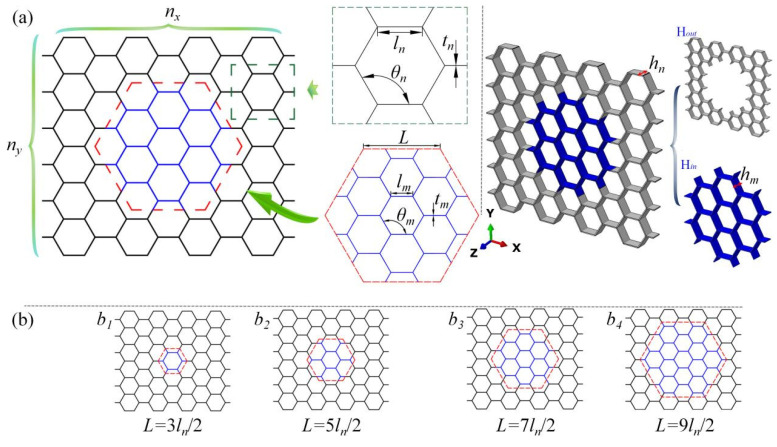
Schematic diagram of modular honeycomb design, (**a**) modular honeycomb design process and geometric parameters, (**b**) modular honeycomb blocks with various edge lengths.

As depicted in Figure 2a, the bio-inspired design emulates the gradient density distribution observed in tree trunks through strategic arrangement of honeycomb cells with differential wall thicknesses in the central and peripheral zones of modular blocks. This configuration achieves radial density gradients analogous to natural arboreal systems. Depending on specific topological requirements, the design allows two distinct optimization approaches: (1) implementing thicker-walled cells in the core region compared to peripheral cells or, conversely, (2) deploying denser cellular arrangements in the peripheral zones relative to the central core. The honeycomb cells located in the central region of the block are termed the sub-honeycomb (Hin). In contrast, the honeycomb cells in the peripheral region are called the matrix honeycomb (Hout). The cell wall length, thickness, height, and angle of the sub-honeycomb are denoted as $l_{n}$, $t_{n}$, $h_{n}$, and $\theta_{n}$, respectively, and those of the matrix honeycomb are $l_{m}$, $t_{m}$, $h_{m}$, and $\theta_{m}$. All geometric parameters, except for wall thickness, are maintained identically to ensure appropriate geometric connectivity between the sub-honeycomb and the matrix honeycomb. Figure 2b presents honeycomb blocks with varying layout patterns, where the edge lengths L of the sub-honeycombs in b1, b2, b3, and b4 are $3l_{n}/2$, $5l_{n}/2$, $7l_{n}/2$, and $9l_{n}/2$, respectively.

**Figure 3:**
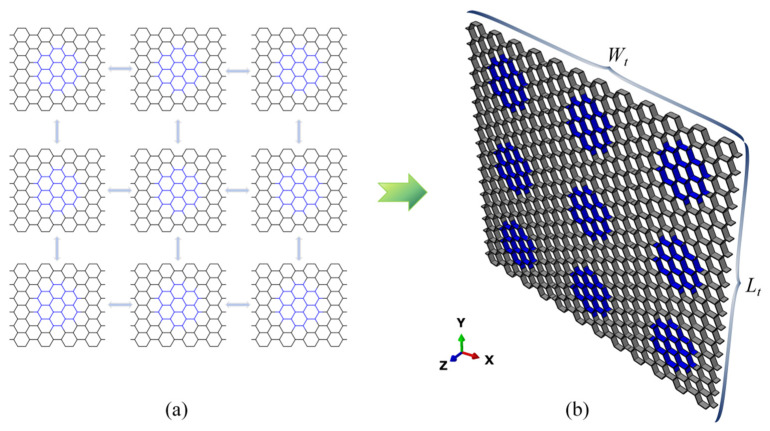
The assembly process of a modular honeycomb, (**a**) assembly process of modular honeycomb structures, (**b**) the modular honeycomb configuration employed in this study.

By assembling $N_{x}\times N_{y}$ honeycomb blocks with the same layout pattern, a modular honeycomb can be constructed, as shown in Figure 3. The overall mechanical performance of modular honeycombs, such as total energy absorption and total load-bearing capacity, is significantly influenced by the parameters $N_{x}$ and $N_{y}$. To ensure comparability across this study, the modular honeycomb illustrated in Figure 3b is uniformly adopted as the benchmark configuration, where $N_{x}$ = 3, $N_{y}$ = 3, and L = $7l_{n}/2$.

### 2.2. Numerical Method

**Figure 4:**
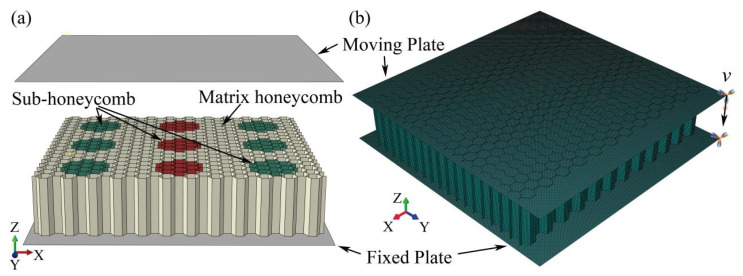
Schematic diagram of numerical simulation of modular honeycomb out-plane compression, (**a**) geometric model, (**b**) mesh model.

Finite element analysis serves as a critical tool for investigating the out-of-plane compressive behavior of honeycombs and is extensively employed in characterizing their deform modes, stress distribution, and energy absorption characteristics under different speed and impact loadings. This study develops a finite element model for modular honeycombs using the ABAQU/CAE/SIMULIA 2023. As illustrated in Figure 4, the geometric model was constructed in ABAQUS, where sub-honeycombs are embedded within a matrix honeycomb and constrained between two rigid plates. The fixed plate remains fully constrained, while the upper moving plate applies controlled velocity impact loading along the *y*-axis. Contact interactions are defined using a general self-contact algorithm for cell wall surfaces and automatic surface-to-surface contact between honeycomb structures and rigid plates, eliminating penetration during simulation. The cellular structure employs aluminum alloy material with elastic modulus of 68.2 GPa, density of 2700 kg/m^3^, and Poisson’s ratio of 0.3. Four-node reduced integration shell elements (S4R) discretize the cell walls. Impact velocity parameters are adjusted through varying impact durations to quantify their effects on deformation patterns and energy absorption efficiency. Relative density control is achieved by modifying cell wall thickness.

**Figure 5:**
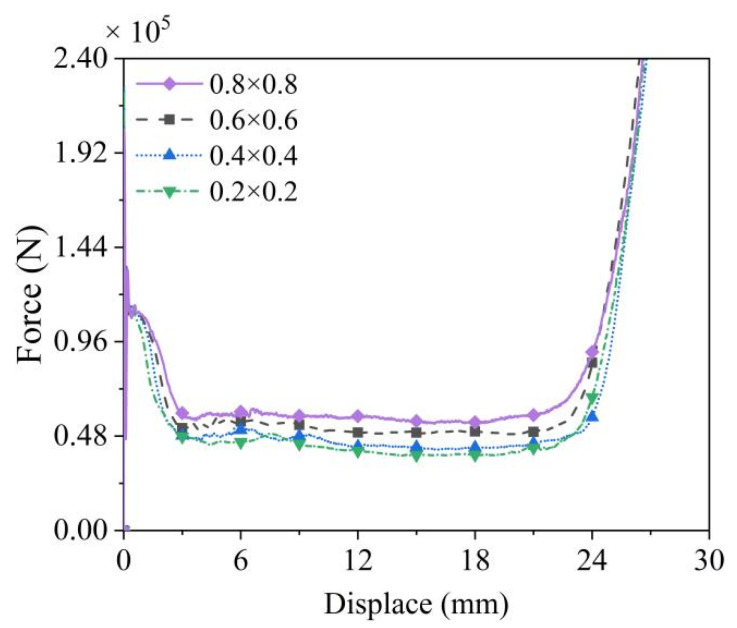
Mesh convergence analysis of the finite element model. Numerical models with mesh dimensions of 0.8 × 0.8, 0.6 × 0.6, 0.4 × 0.4, and 0.2 × 0.2 were evaluated.

To balance computational efficiency and accuracy, mesh convergence analysis was performed to determine the optimal mesh size. Four models with identical parameters were discretized using mesh dimensions of 0.8 × 0.8, 0.6 × 0.6, 0.4 × 0.4, and 0.2 × 0.2, respectively. The force–displacement curves from the 0.4 × 0.4 and 0.2 × 0.2 meshes exhibit negligible differences, whereas results from the 0.8 × 0.8 and 0.6 × 0.6 meshes show significantly higher force values than those of the 0.4 × 0.4 mesh, as shown in Figure 5. Based on these observations, the 0.4 × 0.4 mesh configuration is selected to ensure sufficient accuracy while avoiding excessive computational costs associated with the 0.2 × 0.2 mesh.

## 3. Quasi-Static Compression

This section investigates the mechanical response of modular honeycombs under quasi-static compression conditions through combined theoretical modeling and numerical simulation. The analysis focuses on characterizing plateau stress evolution, revealing how parameter adjustments between sub-honeycomb and matrix honeycomb components enhance load-bearing capabilities compared to conventional designs.

### 3.1. Theoretical Model for Plateau Stress

**Figure 6:**
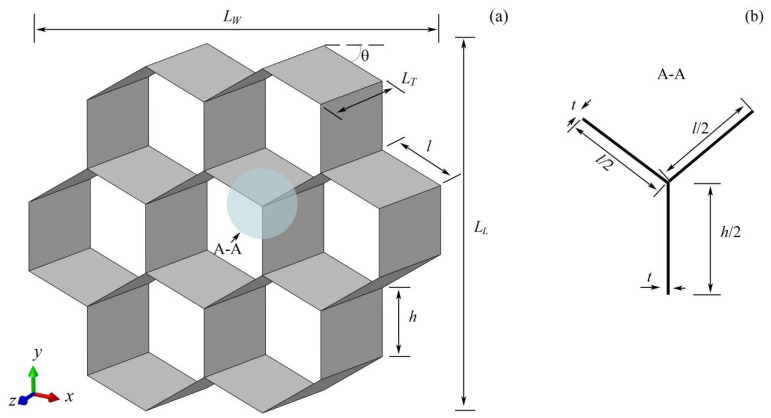
The dimensions of conventional honeycombs (**a**) and the “Y”-shaped energy-absorbing units (**b**). $L_{W}$ is the total width of the honeycomb. $L_{L}$ is the total length of the honeycomb. $L_{T}$ is the depth of the honeycomb. l is the length of the inclined edge. h is the vertical edge height. θ is the angle between the inclined edge and the horizontal line.

The analytical relationship between out-of-plane compressive stress and geometric/material parameters for modular honeycombs is established here. Modular honeycombs are composed of conventional honeycombs with varying wall thicknesses. The theoretical derivation of the out-plane plateau stress for these structures can be approached similarly to that of uniform honeycombs under compression. For descriptive clarity, the geometric dimensions of the sub- and matrix honeycombs in the theoretical derivation are consistently denoted using the symbols presented in Figure 6. Figure 6a can be considered as being composed of the periodic arrangement of the “Y”-shaped units shown in Figure 6b.

**Figure 7:**
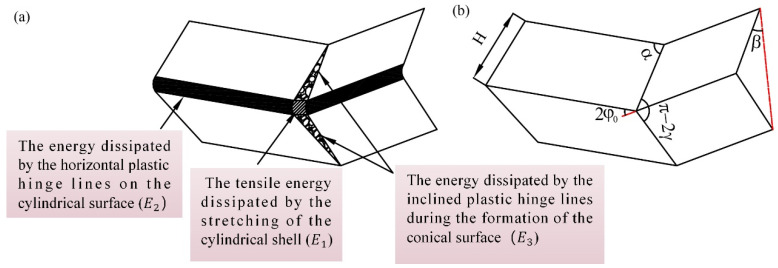
The diagrams of (**a**) the distribution of energy-absorbing units on the honeycomb surface and (**b**) their geometric relationships.

The energy absorption of a single “Y”-shaped folding unit within both the sub- and matrix honeycombs is comprised of three components: the tensile energy dissipated by the stretching of the cylindrical shell ($E_{1}$), the energy dissipated by the horizontal plastic hinge lines on the cylindrical surface ($E_{2}$), and the energy dissipated by the inclined plastic hinge lines during the formation of the conical surface ($E_{3}$), as depicted in Figure 7.

The expression for $E_{1}$ is

(1) $$E_{1}=\frac{16M_{0}Hb_{c}I_{1}\left(\varphi_{0}\right)}{t}$$

where $I_{1}\left(\varphi_{0}\right)$ is a geometric correlation parameter, which can be expressed as

(2) $$I_{1}\left(\varphi_{0}\right)=\frac{\pi }{\left(\pi -2\varphi_{0}\right)\tan\varphi_{0}}\int_{0}^{\beta^{*}}\cos\beta \left[\cos\varphi_{0}-\cos\left(\varphi_{0}+\frac{\pi -2\varphi_{0}}{\pi }\gamma \right)\right]d\beta$$

In the above expressions, $M_{0}$ is the plastic limit bending moment, given by $\sigma_{ys}t^{2}/4$, where $\sigma_{ys}$ is the yield stress, and t is the wall thickness; *H* is defined as half the width of a folding unit; $b_{c}$ denotes the radius of the toroidal surface along the meridional direction; and $\beta^{*}$ represents the contact angle at the fully folded state. For a regular hexagonal honeycomb, $\varphi_{0}=\pi /6$. Under full folding, $I_{1}\left(\pi /6\right)=1.05$.

**Figure 8:**
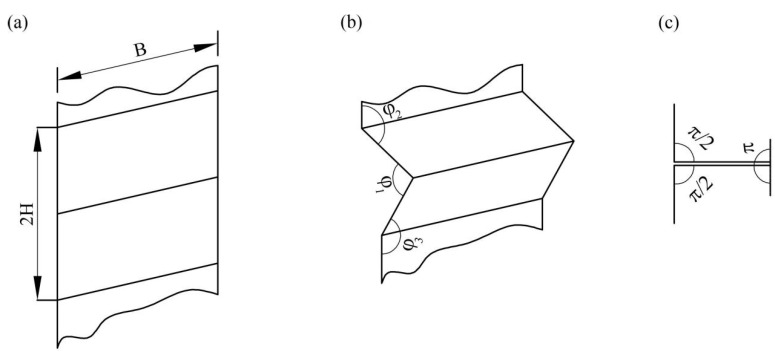
The location and angle of the horizontal hinge line in a fundamental folding unit during the deformation process, (**a**) unfolded state, (**b**) when folded at angle $\varphi_{1}$, (**c**) when folded at angle π.

The illustration in Figure 8 reveals that a primary folding unit within the sub-honeycomb and matrix honeycomb structures undergoes a total folding angle of 2π throughout the deformation process. The following equation can mathematically represent the energy dissipation occurring along the horizontal hinge line:

(3) $$E_{2}=\sum_{i=1}^{3}E_{2i}=2\pi M_{0}\left(l+0.5h\right)$$

$E_{3}$ can be expressed as

(4) $$E_{3}=\frac{{4M_{0}H}^{2}I_{3}\left(\varphi_{0}\right)}{b_{c}}$$

where

(5) $$I_{3}\left(\varphi_{0}\right)=\frac{1}{\tan\phi_{0}}\int_{0}^{\alpha^{*}}\frac{\cos\beta }{\sin\alpha }d\beta$$

in which $\tan\alpha =\tan\varphi_{0}/\sin\beta$. For a hexagonal honeycomb, $I_{3}\left(\pi /6\right)=2.39$. The above derivation indicates that the total energy of the Y-shaped dissipative unit within one wrinkle cycle is

(6) $$E_{Y}=33.6M_{0}\frac{Hb_{c}}{t}+2\pi M_{0}\left(l+0.5h\right)+19.12M_{0}\frac{H^{2}}{b_{c}}$$

Following the principle of energy conservation, the work performed by external forces during the formation of wrinkles is equivalent to the energy dissipated by plastic hinges. Therefore,

(7) $$P_{m}\times 2H=E_{Y}$$

The average load can be obtained by combining Equations (6) and (7). $P_{m}$ is found as follows:

(8) $$P_{m}=M_{0}\left[\frac{16.8}{t}b_{c}+\frac{\pi \left(l+0.5h\right)}{H}+9.56\frac{H}{b_{c}}\right]$$

Based on the equilibrium conditions during axial compression of honeycombs, denoted as $\partial P_{m}/\partial H=0$ and $\partial P_{m}/\partial b_{c}=0$, the values of H and $b_{c}$ can be calculated. The equations for determining the out-of-plane plateau stress $\sigma_{m}$ of the sub-honeycomb and matrix honeycomb structures are as follows:

(9) $$\sigma_{m}\approx 5.2651{\left(\frac{t}{l}\right)}^{\frac{5}{3}}\sigma_{ys}$$

The total reactive force $F_{total}$ experienced by a modular honeycomb under out-plane compression is the resultant force of the plateau stresses of the matrix honeycomb and all sub-honeycombs, which can be expressed as

(10) $$F_{total}=F_{m}+\sum_{n=1}^{N_{x}N_{y}}F_{n}$$

where $F_{m}$ represents the plateau reactive force of the matrix honeycomb and $F_{n}$ represents denotes the plateau reactive force of the *n*th sub-honeycomb, equal to the plateau stress of the sub-honeycomb multiplied by its out-plane cross-sectional area. For a sub-honeycomb with a cell edge length of $l_{n}$ and a wall thickness of $t_{n}$,

(11) $$F_{n}=\frac{15.7953\sqrt{3}}{4}N_{n}{{(t}_{n})}^{\frac{5}{3}}{{(l}_{n})}^{\frac{1}{3}}\sigma_{ys}$$

where $N_{n}$ represents the number of “Y”-shaped energy-absorbing units in the sub-honeycomb. Similarly, for a matrix honeycomb with a cell edge length of $l_{m}$ and wall thickness of $t_{m}$,

(12) $$F_{m}=\frac{15.7953\sqrt{3}}{4}N_{m}{{(t}_{m})}^{\frac{5}{3}}{{(l}_{m})}^{\frac{1}{3}}\sigma_{ys}$$

where $N_{m}$ denotes the number of “Y”-shaped energy-absorbing units within the matrix honeycomb. By substituting the equations above into Equation (10),

(13) $$F_{total}=\frac{15.7953\sqrt{3}}{4}\sigma_{ys}{[N}_{m}{{(t}_{m})}^{\frac{5}{3}}{{(l}_{m})}^{\frac{1}{3}}+\sum_{n=1}^{N_{x}N_{y}}N_{n}{{(t}_{n})}^{\frac{5}{3}}{{(l}_{n})}^{\frac{1}{3}}]$$

The plateau stress of the entire modular honeycomb $\sigma_{ma}$ is

(14) $$\sigma_{ma}=\frac{5.2651\sigma_{ys}{[N}_{m}{{(t}_{m})}^{\frac{5}{3}}{{(l}_{m})}^{\frac{1}{3}}+\sum_{n=1}^{N_{x}N_{y}}N_{n}{{(t}_{n})}^{\frac{5}{3}}{{(l}_{n})}^{\frac{1}{3}}]}{N_{m}{l_{m}}^{2}+\sum_{n=1}^{N_{x}N_{y}}N_{n}{l_{n}}^{2}}$$

For the modular honeycomb shown in Figure 3, $l_{m}=l_{n}$, $N_{n}=24$, with a total of 9 honeycomb blocks in modular honeycomb; therefore,

(15) $$\sigma_{ma}=\frac{5.2651\sigma_{ys}[27{{(t}_{m})}^{\frac{5}{3}}+\sum_{n=1}^{9}{{(t}_{n})}^{\frac{5}{3}}]}{36{{(l}_{n})}^{\frac{5}{3}}}$$

### 3.2. Numerical Verification

To further elucidate the mechanical behavior of modular honeycombs under quasi-static compression, this study employed the finite element method to conduct a detailed numerical analysis, as previously described. Using the numerical methods and Equation (15), the compression plateau stress was calculated for two types of modular honeycombs with different sub-honeycomb wall thicknesses.

**Figure 9:**
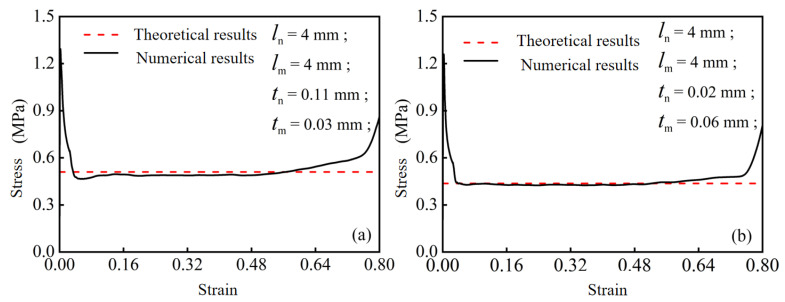
Comparison of numerical and theoretical stress–strain curves for modular honeycombs: (**a**) sub-honeycomb wall thickness 0.03 mm and matrix honeycomb wall thickness 0.11 mm; (**b**) sub-honeycomb wall thickness 0.02 mm and matrix honeycomb wall thickness 0.06 mm.

For the first modular honeycomb configuration, both the sub-honeycomb and matrix honeycomb have a uniform cell edge length of 4 mm. The wall thicknesses are 0.11 mm for the sub-honeycomb and 0.03 mm for the matrix honeycomb. The numerical and theoretical plateau stress values for this configuration are presented in Figure 9a. The stress–strain curve obtained from numerical calculations clearly illustrates the mechanical response under compressive loading. As shown in Figure 9a, when the strain reaches approximately 0.04, the stress enters the plateau stage, stabilizing around 0.5 MPa with minor fluctuations. This value is very close to the theoretical prediction, indicating that the theoretical model can accurately predict the actual mechanical behavior of the modular honeycomb under this configuration.

For the second modular honeycomb, the cell edge length remains at 4 mm for both the sub-honeycomb and matrix honeycomb. However, the wall thicknesses are adjusted to 0.02 mm for the sub-honeycomb and 0.06 mm for the matrix honeycomb. The plateau stress for this configuration is depicted in Figure 9b. As shown in Figure 9b, when the strain reaches approximately 0.04, the stress enters the plateau stage, stabilizing around 0.44 MPa. This value also aligns well with the theoretical prediction, further validating the accuracy of the theoretical model.

Figure 9a,b demonstrate a strong correlation between the theoretical predictions and the numerical results obtained through finite element analysis. Specifically, the stress–strain curves for both configurations show high consistency with the theoretical predictions when entering the plateau stage. This consistency not only validates the reliability of the computational methods employed but also provides a robust methodology for future research on the out-plane performance of modular honeycombs.

Given a traditional honeycomb with a density equivalent to that of the modular configuration, the wall thickness $t_{c}$ lies between the wall thicknesses of the sub- and matrix honeycombs. The edge length of the traditional honeycomb cell is equal to the edge lengths of both the sub-honeycombs and matrix honeycombs. The plateau stress can be determined using the following expression:

(16) $$\sigma_{mc}\approx 5.2651{\left(\frac{t_{c}}{l_{n}}\right)}^{\frac{5}{3}}\sigma_{ys}$$

The ratio of plateau stress between modular honeycomb and conventional honeycomb ζ is

(17) $$\zeta =\frac{{[N}_{m}{{(t}_{m})}^{\frac{5}{3}}+\sum_{n=1}^{N_{x}N_{y}}N_{n}{{(t}_{n})}^{\frac{5}{3}}]}{{(N}_{m}+\sum_{n=1}^{N_{x}N_{y}}N_{n}){\left(t_{c}\right)}^{\frac{5}{3}}}$$

By judiciously adjusting the parameters in Equation (17), specifically the values of $t_{m}$ and $t_{n}$, the dimensionless ratio ζ can be increased beyond unity. This adjustment enables the plateau stress of the modular honeycomb to surpass that of a conventional honeycomb of equivalent density. Consequently, the strategic manipulation of these parameters in Equation (17) not only demonstrates the potential for modular honeycombs to achieve higher plateau stress values compared to their traditional counterparts but also elucidates the underlying enhancement mechanism. This theoretical insight provides a comprehensive explanation for the superior mechanical performance of modular honeycombs, highlighting the significance of parameter optimization in achieving enhanced load-bearing capabilities.

## 4. High-Speed Crushing

The dynamic compressive behavior under high-speed impact conditions is systematically investigated here, extending the quasi-static analysis in Section 3 to crushing velocity spanning 20–100 m/s. Three modular honeycomb groups with relative densities of 1.44–3.46% are examined. Particular emphasis is placed on quantifying velocity-dependent plateau stress enhancement and its correlation with energy absorption characteristics through parametric comparisons.

### 4.1. Plateau Stress

To investigate the mechanical response of modular honeycombs under out-of-plane high-velocity impact, three groups with relative densities of 1.44%, 2.31%, and 3.46% are strategically configured, as detailed in Table 1, where $M_{n}$ and $M_{m}$ denote the respective masses of the sub-honeycomb and matrix honeycomb. The plateau stresses and specific loads of distinct modular honeycombs are numerically determined using the aforementioned computational methodology.

**Figure 10:**
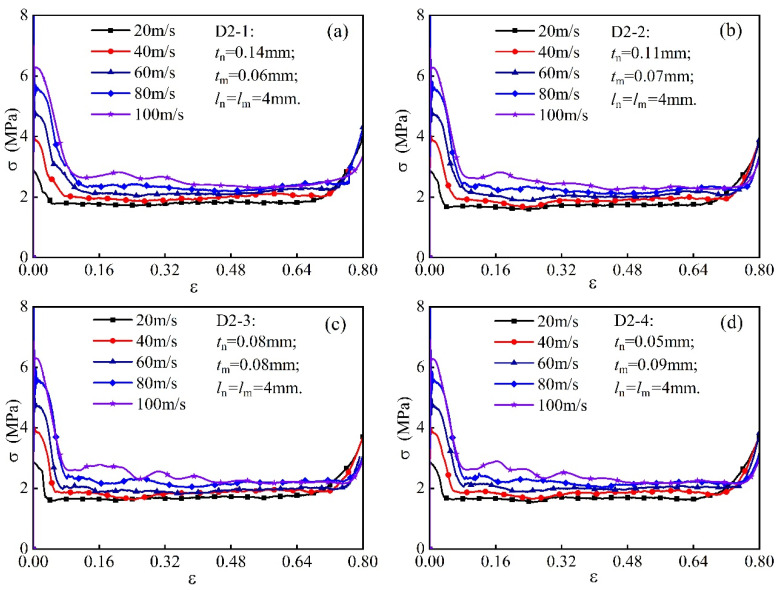
Stress–strain curves of honeycombs at different impact velocities, (**a**) D2-1, (**b**) D2-2, (**c**) D2-3, (**d**) D2-4.

Groups D1 to D3 in Table 1 each comprise four types of modular honeycombs with varying wall thicknesses, wherein the sub-honeycomb wall thickness decreases, and the matrix honeycomb wall thickness increases. The honeycomb structure under investigation demonstrates consistent cell wall dimensions of 4 mm across all specimens, with the entire assembly measuring 30 mm in height. Plateau stress behavior is analyzed at five specific velocities ranging from 20 m/s to 100 m/s, with increments of 20 m/s between each measurement point. Figure 10a–d present the stress–strain curves of the four modular honeycombs in group D2 at varying impact velocities. As impact velocity increases, the modular honeycomb structures exhibit a higher plateau stress. At an impact velocity of 20 m/s, all honeycomb structures exhibit stress–strain curves characterized by minimal fluctuations in a relatively uniform pattern. As the velocity increases, the stress of the honeycombs increases progressively, and the stress fluctuations with strain become more pronounced. The impact process demonstrates a notable correlation between impact velocity and the temporal occurrence of the densification point. As the impact velocity increases from 20 m/s to 100 m/s, a substantial delay in the onset of densification is observed, with the densification point occurring markedly later in the higher-velocity scenario. The investigation demonstrates that the plateau stress of modular honeycombs exhibits an increase with elevated impact velocities, concomitant with more pronounced stress fluctuations and a posterior displacement of the densification point.

**Figure 11:**
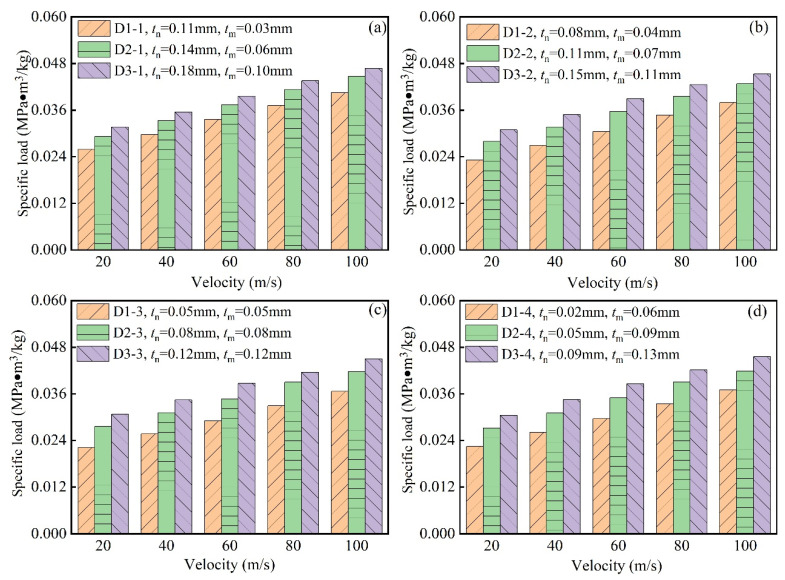
Specific load of honeycombs with different relative densities under impact, (**a**) D1-1, D2-1 and D3-1, (**b**) D1-2, D2-2 and D3-2, (**c**) D1-3, D2-3 and D3-3, (**d**) D1-4, D2-4 and D3-4.

Figure 11a–d systematically elucidate the dynamic load-bearing characteristics of modular honeycomb structures under impact velocities spanning 20–100 m/s with varied wall-thickness configurations. The specific load, defined as the ratio of plateau stress to relative density under compressive impact, serves as the key performance metric. Figure 11a comparatively analyzes three modular configurations (D1-1, D2-1, D3-1), demonstrating progressive enhancement of specific load with increasing impact velocity (20 → 40 → 60 → 80 → 100 m/s). Concurrently, the ascending relative density gradient (D1-1: 1.44% < D2-1: 2.31% < D3-1: 3.46%) induces elevation of specific load at equivalent velocities.

Figure 11b,d confirm this correlation between impact velocity, relative density, and specific load across supplementary modular configurations. The conventional homogeneous-walled counterparts in Figure 11c exhibit similar trends. However, comparative analysis of Figure 11b,d reveals superior performance of modular designs: at matched relative densities and velocities, modular configurations demonstrate higher specific loads than their conventional counterparts. This mechanical enhancement conclusively verifies the proposed modular architecture’s capacity for elevated impact energy absorption through optimized cell wall redistribution.

### 4.2. Specific Energy Absorption

**Figure 12:**
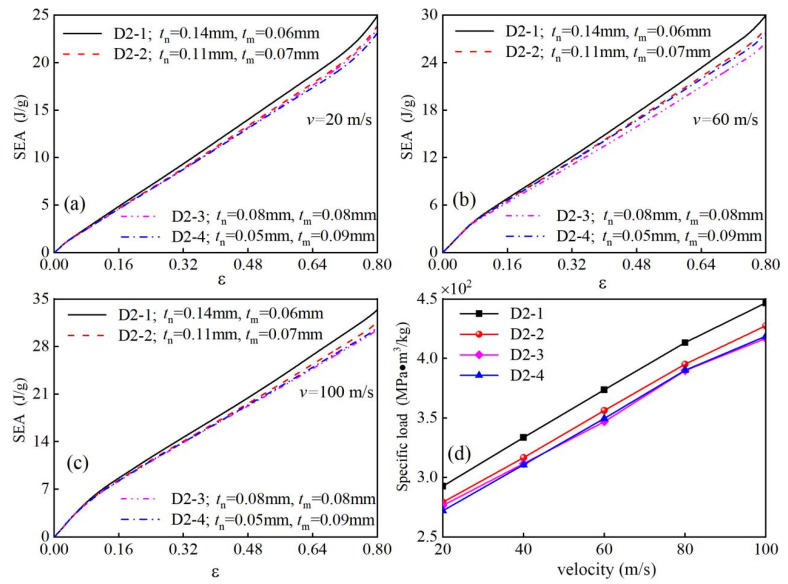
The specific energy absorption curves of D2-1, D2-2, D2-3, and D2-4 honeycombs at impact velocities of 20 m/s (**a**), 60 m/s (**b**), and 100 m/s (**c**) and the specific load of each honeycomb at different impact velocities (**d**).

To quantitatively compare the energy absorption performance of modular honeycombs at equivalent relative densities, Figure 12a–c systematically presents the specific energy absorption (SEA) characteristics of D2-1, D2-2, D2-3, and D2-4 configurations under impact velocities of 20 m/s, 60 m/s, and 100 m/s. The data demonstrate the superior performance of the bio-inspired modular design: D2-1 achieves the highest SEA values across all tested velocities, exhibiting 5.82%, 10.56%, and 8.97% enhancements compared to the conventional homogeneous-walled D2-3 configuration at 20 m/s, 60 m/s, and 100 m/s, respectively. This verifies that the modular honeycomb design improves specific energy absorption efficiency over traditional honeycombs under out-of-plane impact. Furthermore, Figure 12a–c reveal that all honeycomb configurations exhibit significant SEA enhancement with increasing impact velocity. Taking D2-1 as an example, the SEA reach 24.9 J/g, 29.85 J/g, and 33.29 J/g at impact velocities of 20 m/s, 60 m/s, and 100 m/s, respectively. This indicates a positive correlation between the energy absorption capacity of modular honeycombs and impact velocity, a characteristic shared with traditional honeycomb structures.

Figure 12d illustrates the variation trends of specific load with increasing velocity for different honeycomb configurations. The results reveal a consistent gradual increase in the specific load for all honeycomb types as impact velocity escalates, indicating a positive correlation between specific load and impact intensity—higher velocities correspondingly enhance structural load-bearing capacity. Notably, the modular honeycomb D2-1 demonstrates superior performance by maintaining the highest specific load across all tested velocities compared to the other three configurations. Specifically, when benchmarked against the conventional uniform-walled D2-3 configuration, the D2-1 design achieves significant enhancements of 5.80%, 7.07%, 7.78%, 5.92%, and 7.19% at velocities of 20 m/s, 40 m/s, 60 m/s, 80 m/s, and 100 m/s, respectively. This systematic improvement quantitatively verifies that the modular design strategy effectively optimizes the load-bearing capability of honeycomb structures under dynamic loading conditions.

## 5. Discussion

The design of modular honeycombs for impact-resistant applications requires systematic investigation of two critical factors: the thickness matching between sub-honeycomb and matrix honeycomb and the boundary effects arising from inter-cell connections. This section examines how the wall-thickness difference between sub-honeycombs and matrix honeycombs governs progressive collapse patterns under quasi-static compression while simultaneously evaluating how boundary discontinuities between adjacent honeycombs influence structural integrity and energy dissipation capacity.

### 5.1. Matching Effect Under the Quasi-Static Compression

Four modular honeycombs with a relative density of 1.73% are designed in Table 2 to investigate the influence of the matching relationship between the wall thickness of the sub- and matrix cells in the modular honeycomb on its impact resistance characteristics. The wall thickness of the sub-honeycomb in BJ2-m-0.04 and BJ2-m-0.05 is greater than that of the matrix honeycomb, while the wall thickness of the sub-honeycomb in BJ2-m-0.06 is equal. The wall thickness of the sub-honeycomb in BJ2-m-0.07 is less than that of the matrix honeycomb. The height of the sub- and matrix cells in each honeycomb is 30 mm, and the length of the cell wall is 4 mm. Using numerical methods to model and analyze four types of honeycomb structures, we explored the relationship between wall-thickness matching of matrix honeycomb and sub-honeycombs and the deformation mode and mechanical properties of modular honeycomb.

**Figure 13:**
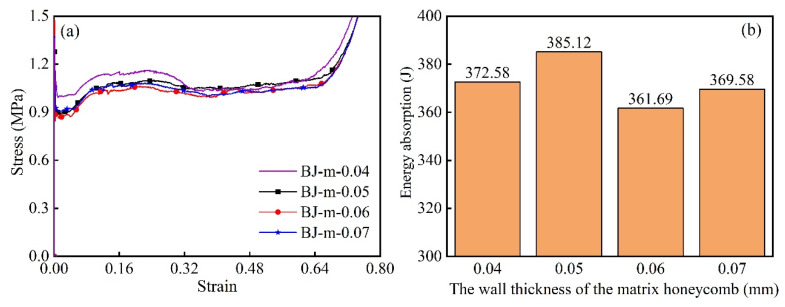
The mechanical performance of four types of modular honeycomb with the same relative density: (**a**) stress–strain curve; (**b**) energy absorption.

Figure 13a shows the stress–strain curve of the modular honeycombs in Table 2 under quasi-static compression impact. The curves show that the stress of BJ2-m-0.04 and BJ2-m-0.05 is higher than that of the traditional honeycomb (BJ2-m-0.06). The stress–strain curve of BJ2-m-0.07 is higher than BJ2-m-0.06 in the early stage and equal to BJ2-m-0.06 in the later stage. Figure 13b illustrates the energy absorption capacity of various honeycomb structures up to their compaction point. The data demonstrate that modular honeycomb configurations consistently outperform uniform honeycomb designs in terms of total energy absorption. BJ2-m-0.05 has increased by 6.48% compared to BJ2-m-0.06, with the most significant improvement effect. A comparison of Figure 13a,b indicates that BJ2-m-0.04 demonstrates higher stress values than BJ2-m-0.05 during the initial compression phase, while their stress levels become comparable in the later stages.

Nevertheless, the overall energy absorption capacity of BJ2-m-0.04 is less than that of BJ2-m-0.05. This is due to the thicker cell wall of the BJ2-m-0.04, which leads to its early entry into the compaction stage and insufficient energy absorption of the matrix honeycomb. The observed phenomenon demonstrates that an appropriate balance between the wall thicknesses of the matrix and sub-honeycomb structures contributes significantly to maximizing the impact resistance capabilities of the modular honeycomb configuration.

**Figure 14:**
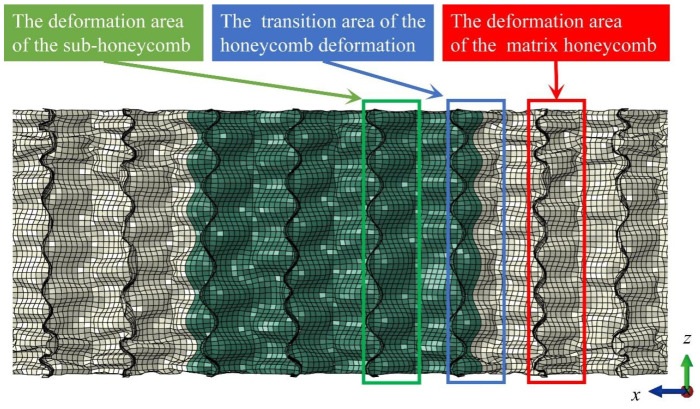
Deformation mode of BJ2-m-0.04 modular honeycomb under the strain of 0.36.

To elucidate the underlying mechanism by which the correspondence between wall thicknesses of the matrix and sub-honeycomb structures influences the impact resistance characteristics of the modular honeycomb, Figure 14 illustrates the deformation pattern exhibited by honeycomb BJ2-m-0.04 when subjected to a strain of 0.36. The modular honeycomb deformation zone is divided into a sub-honeycomb deformation zone, a matrix honeycomb deformation zone, and a transition zone at the connection between the two. The deformation modes of each region in the modular honeycomb demonstrate a strong correlation with the wall thickness of the sub-honeycomb and the matrix honeycomb, exhibiting the following general characteristics. Firstly, the quantity of deformation folds on thin cell walls significantly exceeds that on thick cell walls, as illustrated in Figure 13, where the sub-honeycomb wall thickness is 0.12 mm, and the matrix honeycomb wall thickness is 0.04 mm. The matrix honeycomb folds observed in Figure 14 are substantially more pronounced than those on the sub-honeycomb walls. The second observation is that the quantity of folds on the cell wall in the transition zone is equivalent to the number of folds on the thick cell wall, as illustrated in Figure 14. The number of folds and the mode of deformation exhibited by the cell wall within the transition zone are identical to those observed in the sub-honeycomb structure. The observed phenomenon demonstrates that a considerable variation in wall thickness between the sub- and matrix honeycomb structures can inhibit the formation of thin cell wall folds within the transition zone, consequently reducing the energy absorption capabilities of the thin-walled honeycomb configuration. This crucial aspect is a primary factor contributing to the enhanced energy absorption efficiency of BJ2-m-0.05 compared to BJ2-m-0.04.

### 5.2. Boundary Effects Under the Quasi-Static Compression

When there are connected defects between sub-honeycombs and the matrix honeycomb, the circumferential edges of both can become disordered under the out-plane impact, leading to a decrease in the impact resistance of the honeycomb. This phenomenon is referred to as the boundary effect in modular honeycombs. In this section, numerical methods are employed to thoroughly analyze the relationship between the boundary effect of modular honeycombs and factors such as the thickness of sub-honeycomb walls, the layout of honeycomb blocks, and the length of the edges.

To investigate the impact of sub-honeycomb wall thickness on boundary effects, five sets of modular honeycombs, designated as B1 to B5, with varying wall thicknesses were designed, as shown in Table 3. Within each set, the sub- and matrix honeycombs have equal wall thicknesses. The letters “C” and “U” in the designation represent the fixed and independent connected states of the sub- and matrix honeycombs, respectively. The wall thickness of the matrix honeycomb in all sets remains constant at 0.06 mm, while the wall thickness of the sub-honeycomb increases incrementally by 0.02 mm from 0.02 mm to 0.10 mm. The dimensions of the modular honeycombs comprise a 30 mm height and a 4 mm cell wall length. A tabular representation delineates the wall thickness and mass for sub- and matrix honeycombs across various configurations, as shown in Table 3.

**Figure 15:**
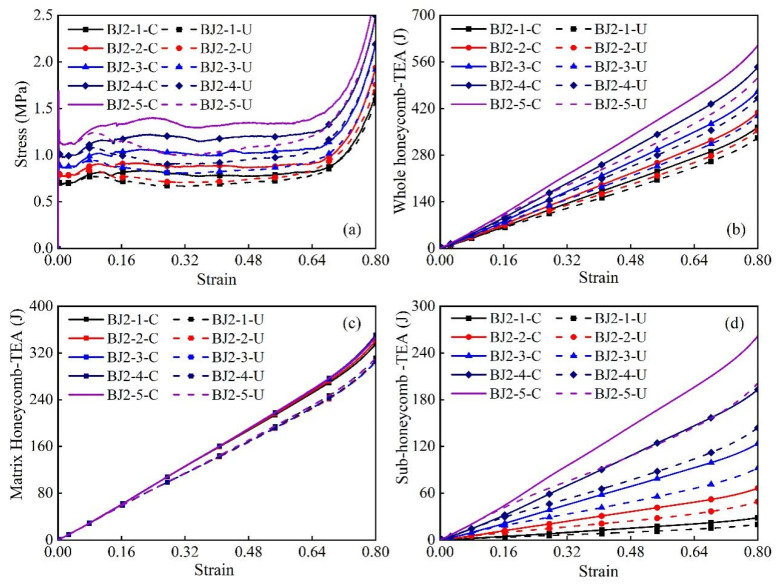
The stress–strain curves (**a**), overall energy absorption (**b**), matrix honeycomb energy absorption (**c**), and sub-honeycomb energy absorption (**d**) of modular honeycombs with varying sub-honeycomb wall thicknesses.

The stress and total energy absorption (TEA) characteristics of the overall modular honeycomb, matrix honeycomb, and sub-honeycomb are presented in Figure 15a–d as functions of compressive strain for the five honeycomb sets under investigation. It can be observed from Figure 15a,b that the plateau stress and total energy absorption within the same group when the sub- and matrix honeycombs are rigidly connected are significantly higher than when independently connected. This confirms that boundary effects can substantially reduce the impact resistance of modular honeycombs.

Further analysis of the curves in Figure 15a reveals that when the matrix honeycomb wall thickness remains constant, and the sub-honeycomb wall thickness increases from 0.02 mm to 0.10 mm, the difference in plateau stress between two connected honeycombs within the same group exhibits a significant increase. Using a sub-honeycomb wall thickness of 0.02 mm as a comparative baseline, an increase of 0.1 mm in the sub-honeycomb wall thickness results in a 70.27% increase in the plateau stress of the rigidly connected modular honeycomb. However, the independently connected modular honeycomb exhibits only a 55.32% increase. A similar phenomenon can be observed in Figure 15b, where the total energy absorption of the rigidly connected modular honeycomb increases by 65.98% as the sub-honeycomb wall thickness grows from 0.02 mm to 0.10 mm. However, the plateau stress of the independently connected modular honeycomb only increases by 53.16%. These observations suggest that an increase in sub-honeycomb wall thickness results in more pronounced boundary effects of the modular honeycomb, consequently leading to a more substantial reduction in impact resistance.

To delve into the mechanism by which boundary effects are enhanced with varying sub-honeycomb wall thickness, Figure 15c,d depict the energy absorption of all matrix and sub-honeycombs during compression, as detailed in Table 3. Figure 15c reveals that the energy absorption of all rigidly connected matrix honeycombs is essentially uniform, as is that of all independently connected matrix honeycombs. Nevertheless, when matrix honeycomb wall thickness is identical, the energy absorption capacity of rigidly connected matrix honeycombs significantly exceeds that of independently connected structures, suggesting that boundary effects substantially diminish the energy absorption of the matrix honeycomb within the modular honeycomb. The magnitude of this reduction demonstrates no significant correlation with variations in sub-honeycomb wall thickness.

Figure 15d contrasts the energy absorption of sub-honeycombs within various connected modular honeycombs. The energy absorption of sub-honeycombs in the rigidly connected state is substantially higher than that in the independent state, and this difference increases as the sub-honeycomb wall thickness increases. When the equivalent strain reaches 0.8, and the sub-honeycomb wall thickness is 0.1 mm, the energy absorption of the rigidly connected sub-honeycomb is 60.93 J greater than that of the independently connected sub-honeycomb, whereas, at a sub-honeycomb wall thickness of 0.02 mm, this difference is only 8.49 J. These observations suggest that changes in sub-honeycomb wall thickness primarily influence the boundary effects of the modular honeycomb by altering its impact resistance rather than that of the matrix honeycomb.

**Figure 16:**
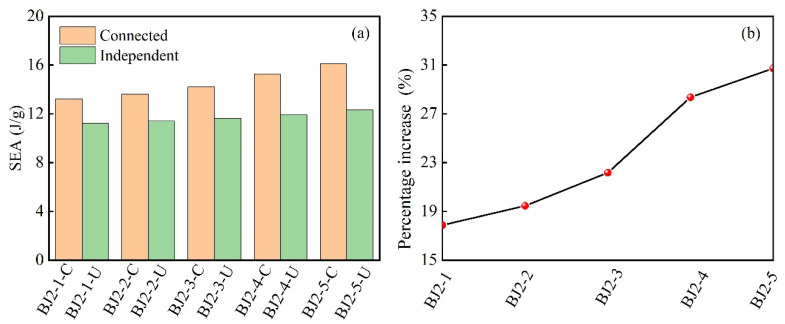
The specific energy absorption (**a**) and specific energy absorption enhancement ratio (**b**) of modular honeycombs in connected and independent states of sub-honeycombs and matrix honeycombs.

Figure 16a presents a comparative analysis of the specific energy absorption of the five sets of modular honeycombs enumerated in Table 3, demonstrating that the specific energy absorption of rigidly connected modular honeycombs substantially exceeds that of independently connected specimens. As the sub-honeycomb wall thickness increases, the specific energy absorption of independently connected modular honeycombs remains almost constant. In contrast, the number of rigidly connected modular honeycombs gradually increases, demonstrating a noticeable improvement in energy absorption efficiency. The enhancement ratio of specific energy absorption is defined as the proportion by which the specific energy absorption of rigidly connected honeycombs exceeds that of independently connected ones within the same group.

Figure 16b plots the enhancement ratio of specific energy absorption for the five sets of honeycombs. The curve rises with the increase in sub-honeycomb wall thickness. The particular energy absorption enhancement ratio of BJ2-1-C compared to BJ2-1-U is approximately 17.9%, while that of BJ2-5-C compared to BJ2-5-U reaches 30.8%. These findings underscore the significant impact of boundary effects on the energy absorption efficiency of modular honeycombs, with the rigid connection leading to a substantial increase in performance as the sub-honeycomb wall thickness grows.

## 6. Conclusions

This study presents a methodology for enhancing honeycomb structures’ out-of-plane load-bearing capacity and energy absorption. A geometric configuration of the modular honeycomb has been constructed, and the mechanisms of load-bearing and energy absorption enhancement have been explored through theoretical analysis and numerical simulation. Boundary and matching effects on the modular honeycomb have also been analyzed. The main conclusions from this research include the following:

(1) The theoretical model for the out-of-plane plateau stress of the modular honeycomb, derived from the principle of energy conservation between the work performed by external forces and the total energy absorbed by plastic hinges, demonstrates strong concordance with the numerical model and can be utilized to predict the out-of-plane mechanical performance of the modular honeycomb. Mathematical analysis of the theoretical model reveals that the enhanced compressive strength of modular honeycombs under quasi-static compression, when compared to uniform-wall-thickness counterparts, stems from the superior load-bearing capacity conferred by thick-walled sub-honeycombs;
(2) Modular honeycomb configurations surpass traditional honeycomb structures of equivalent density in out-of-plane load-bearing capacity and energy absorption. This superiority is consistently observed across impact velocities of 20 m/s, 60 m/s, and 100 m/s, with the modular honeycomb D2-1 exhibiting 5.8%, 7.78%, and 7.19% higher specific loads, along with 5.82%, 10.56%, and 8.97% greater energy absorption than conventional counterparts, thereby demonstrating the mechanical performance advantages in modular design;
(3) Adjusting the wall thickness of sub- and matrix honeycombs can optimize the impact resistance of modular honeycombs. A proper wall-thickness matching relationship prevents the shifting of densification points, the reduction of energy-absorbing wrinkles in the transition zone, and instability, thereby fully leveraging the enhanced impact resistance of modular honeycombs;
(4) The boundary effect can decrease the mechanical performance of modular honeycombs, and it should be limited in practical applications.

## Figures and Tables

**Table 1:** Parameters of modular honeycomb with different relative densities.

Group	Serial Number	Sub-Honeycomb		Matrix Honeycomb		Relative Density
		$t_{n}$/mm	$M_{n}$/g	$t_{m}$/mm	$M_{m}$/g	
D1	D1-1	0.11	11.55	0.03	9.45	1.44%
	D1-2	0.08	8.40	0.04	12.60	
	D1-3	0.05	5.25	0.05	15.75	
	D1-4	0.02	2.10	0.06	18.90	
D2	D2-1	0.14	14.70	0.06	18.90	2.31%
	D2-2	0.11	11.55	0.07	22.05	
	D2-3	0.08	8.40	0.08	25.19	
	D2-4	0.05	5.25	0.09	28.34	
D3	D3-1	0.18	18.90	0.10	31.49	3.46%
	D3-2	0.15	15.75	0.11	34.64	
	D3-3	0.12	12.60	0.12	37.79	
	D3-4	0.09	9.45	0.13	40.94	

**Table 2:** Parameters of modular honeycombs with a relative density of 1.73%.

Serial Number	Layout Form	Sub-Honeycomb		Matrix Honeycomb		Relative Density
		$t_{n}$/mm	$M_{n}$/g	$t_{m}$/mm	$M_{m}$/g	
BJ2-m-0.04	BJ2	0.12	12.6	0.04	12.60	1.73%
BJ2-m-0.05		0.09	9.45	0.05	15.75	
BJ2-m-0.06		0.06	6.30	0.06	18.90	
BJ2-m-0.07		0.03	3.15	0.07	22.05	

**Table 3:** The structural parameters of modular honeycombs with varying sub-honeycomb wall thicknesses and different connected modes.

Group	Serial Number	Connection State	Sub-Honeycomb		Matrix Honeycomb	
			$t_{n}$	$M_{n}$/g	$t_{m}$/mm	$M_{m}$
B1	BJ2-1-C	connected	0.02	2.10	0.06	18.90
	BJ2-1-U	independent	0.02	2.10	0.06	18.90
B2	BJ2-2-C	connected	0.04	4.20	0.06	18.90
	BJ2-2-U	independent	0.04	4.20	0.06	18.90
B3	BJ2-3-C	connected	0.06	6.30	0.06	18.90
	BJ2-3-U	independent	0.06	6.30	0.06	18.90
B4	BJ2-4-C	connected	0.08	8.40	0.06	18.90
	BJ2-4-U	independent	0.08	8.40	0.06	18.90
B5	BJ2-5-C	connected	0.10	10.50	0.06	18.90
	BJ2-5-U	independent	0.10	10.50	0.06	18.90

## Data Availability

The original contributions presented in this study are included in the article. Further inquiries can be directed to the corresponding author.
